# Preparedness for delivering non-communicable disease services in primary care: access to medicines for diabetes and hypertension in a district in south India

**DOI:** 10.1136/bmjgh-2017-000519

**Published:** 2018-01-03

**Authors:** Maya Annie Elias, Manoj Kumar Pati, Praveenkumar Aivalli, Bhanuprakash Srinath, Chikkagollahalli Munegowda, Zubin Cyrus Shroff, Maryam Bigdeli, Prashanth N Srinivas

**Affiliations:** 1 Health equity & evaluation cluster, Institute of Public Health, Bangalore, Karnataka, India; 2 Alliance for Health Policy and Systems Research, WHO, Geneva, Switzerland; 3 Department of Health Systems Governance and Financing, WHO, Geneva, Switzerland

**Keywords:** health services research, diabetes, hypertension

## Abstract

**Introduction:**

Non-communicable diseases (NCDs) have become a major public health challenge worldwide; they account for 28 million deaths per year in low-and-middle-income countries (LMICs). Like many other LMICs, India is struggling to organise quality care for a large NCD-affected population especially at the primary healthcare level. The aim of this study was to assess local health system preparedness in a south Indian primary healthcare setting for addressing diabetes and hypertension.

**Methods:**

This paper draws on a mixed-methods research study on access to medicines conducted in Tumkur, Karnataka, India. We used quantitative data from household and health facility surveys, and qualitative data from focus group discussions and in-depth interviews with health workers and patients. We identified systemic drivers that influence utilisation of services at government primary health centres (PHCs) using thematic analysis of qualitative data and a systems framework on access to medicines to assess supply and demand side factors.

**Results:**

Majority of households depend on private facilities for diabetes and hypertension care because of the lack of laboratory facilities and frequent medicine stockouts at PHCs. Financial and managerial resource allocation for NCDs and prioritisation of care and processes related to NCDs was suboptimal compared to the prominence of this agenda at global and national levels. Primary healthcare has a limited role even in the activities under the national programme that addresses diabetes and hypertension.

**Discussion:**

The study finds critical gaps in the preparedness of PHCs and district health systems in organising and managing care for diabetes and hypertension. Due to the lack of continuous care organised through PHCs, patients depend on expensive and often episodic care in the private sector. There is a need to improve managerial and financial resource allocation towards diabetes and hypertension (and other NCDs) at the district level.

**Trial registration number:**

CTRI/2015/03/005640; Pre-results.

**Key questions**

**What is already known about this topic?**

- There has been widespread global and national attention over the last decade towards strengthening health systems for providing care for non-communicable diseases (NCDs), especially at the primary healthcare level.
- Health systems in low-and-middle-income countries are struggling to organise and manage care for diabetes and hypertension within their public health systems.
- India has launched a national programme to address several NCDs including diabetes and hypertension in 2010.

**What are the new findings?**

- Despite global and national focus on care for NCDs, local health systems are yet to prioritise action at primary and secondary care levels.
- The study examines private and public sector interactions in the area of diabetes and hypertension care and reports on their effects on care in a mixed health systems setting such as in India.
- The study found gaps in infrastructure, human resource availability, performance as well as governance of local health systems, which could explain private sector dependence of patients with diabetes and hypertension.

**Recommendations for policy**

- There is a need for local health system planners and managers to prioritise care for diabetes and hypertension.
- There is a need to invest in infrastructure, in improving the availability and performance of human resources as well as in improving the credibility of government primary healthcare systems.
- Health system regulations need to address irrational treatment practices for diabetes and hypertension and improve coordination between public and private sectors.

## Introduction

Non-communicable diseases (NCDs) have become a major public health challenge worldwide. In low-and-middle-income countries (LMICs), they account for 28 million deaths per year, almost three-quarters of global NCD deaths.^1^ Cardiovascular diseases, cancers, respiratory diseases and diabetes are the most common NCDs, which result in high mortality rates and associated disabilities;^2^ the economic burden and disabilities associated with NCDs have become the subject of policy debate globally.^3^ The global economic burden of NCDs is estimated to rise to $13 trillion by 2030. Every 10% increase in NCDs is expected to be associated with a 0.5% decrease of annual economic growth at the global level.^1 4^ The 2012 World Health Assembly highlighted NCDs as a major challenge and proposed various strategies including a global monitoring framework and strengthening of NCD policies.^5^

India is struggling to organise quality care for a large NCD-affected population with 60% of all deaths in the country attributed to NCDs.^6^ Cardiovascular diseases account for a majority of these deaths followed by cancer and diabetes, which marks a significant shift in India’s epidemiological profile over the past few decades when communicable diseases accounted for a majority of deaths. In India, expenditure on NCD care is associated with poverty.^7^ One in every four families with a cardiovascular disease faces catastrophic health expenditure and 10% of these families fall into poverty, often distorting household priorities such as education of children and housing.^7^ However, India’s public healthcare system, especially at the primary care level has continued to largely focus on communicable diseases almost to the exclusion of NCDs which have often wrongly been perceived as diseases of affluence.^8^

At global and national levels however, the acknowledgement of the problem has been evident; India was one of the first countries in the world to adopt the global monitoring framework on NCDs and develop specific national targets and indicators aimed at reducing the number of global premature deaths from NCDs by 25% by 2025, along the lines of WHO’s Global Action Plan for the Prevention and Control of NCDs.^9^ In 2008, India launched the National Programme for the Prevention and Control of Cancer, Diabetes, Cardiovascular Disease and Stroke (NPCDCS).^10^ The programme highlights the need for a comprehensive management strategy for NCDs, including risk reduction for prevention, early diagnosis, appropriate management and specific intervention strategies for different levels of health services. However, allocation of resources or activities to address NCDs at the primary healthcare level is minimal, whereas the focus remains on identifying activities for NCD care and prevention at secondary and tertiary care facilities.

Access to and utilisation of medicines is one of the foundations on which a responsive health system is built; for NCDs such as diabetes and hypertension, access to continuous care and treatment at a nearby primary care facility is crucial.^11^ The WHO Global Action Plan and Monitoring Framework for NCDs proposed an 80% availability of affordable basic technologies and essential medicines including generics, that are required to treat major NCDs in both government and private facilities.^12^ However, in India, medicines account for more than 70% of out-of-pocket (OOP) healthcare expenditure (expenses on healthcare (including medicines and other ancillary services) directly paid for by the patient at the time of service delivery). This is often due to the lack of availability of medicines in government health facilities despite a mandate to provide medicines free of cost.^13 14^

The last decade of health reforms (such as the National Health Mission) have focused on strengthening decentralised planning and management to the district level. Most districts in India are quite large (in terms of population ranging from 1,000,000 to 5,000,000 people). They are an important unit for organisation and management of care. One of the key strategies for strengthening health systems in India would be to improve management and leadership at the district level. Districts are also the point of interface between vertical disease control programmes and more demand-driven agendas of the local population, and are hence the ideal level at which to assess health system preparedness.

This paper is part of a mixed-methods study of a health system intervention for improving equitable access to medicines for diabetes and hypertension in Karnataka carried out from 2013 to 2016.^15^ In this paper, we use the Access to Medicines framework developed by Bigdeli *et al* 16 to assess health system preparedness for addressing diabetes and hypertension at the primary health centre (PHC) level in a south Indian district. We do this through an assessment of supply and demand side factors that influence utilisation of services at government PHCs for diabetes and hypertension using data from quantitative surveys, focus group discussions (FGDs) and in-depth interviews.^15^

## Methods

### Study setting

Karnataka is one of five south Indian states, and has a population of 61 million. With an infant mortality rate of 31 per 1,000 live births (national average 40), maternal mortality ratio of 144 per 100 000 live births (national average 178) and a female literacy rate of 68% (national average 65.5), several health and development indicators of Karnataka are only slightly better off than the national average.^17–19^ The state has 30 districts. District populations range from 500 000 in Kodagu district to nearly 10 million in Bangalore urban district.^19^ Tumkur is a district with 10 *talukas* (administrative subdivision of districts), with a total population of 2.6 million, of which 3 *talukas* were included in the study.

### Data collection

The quantitative component of the study included a household survey using a cluster-randomised design. In addition, a facility survey of government PHCs and private pharmacies, and exit interviews with patients obtaining care at PHCs were also conducted. The household survey focused on the health-seeking behaviour of patients with diabetes and hypertension, their socioeconomic status, perceptions of price, quality, and access to and utilisation of medicines. A total of 107 clusters were identified based on differing distances from the PHC;^i^ in each cluster, we surveyed an average of 10 households from (1) within a 5 km distance from the PHC (cluster a), (2) between 5 km and 10 km (cluster b), and (3) more than 10 km distance from the PHC (cluster c). Households with at least one member with hypertension or diabetes were included in the survey. In PHCs which catered to smaller populations, we could get only one or two clusters instead of three. Similarly in the case of villages with small populations where 10 households with diabetes and hypertension were not found, the survey extended to the nearest village. The survey covered a total of 1069 households with 1157 patients with either hypertension or diabetes or both. The total number of patients considered for final analysis and interpretation was 1149 (excluding children and adolescents with type 1 diabetes as the focus of the study was on adult-onset type 2 diabetes).

The facility survey was conducted in 39 PHCs and 30 private pharmacies in the study *talukas*. This survey captured the availability of key essential medicines for diabetes and hypertension and their stockout duration in the previous year. We looked at the record-keeping for medicines, their storage conditions and handling, patient care and prescribing practices of primary care doctors, and availability of standard treatment guidelines and essential medicines lists in PHCs. The exit interviews of patients with diabetes and hypertension captured the average number of dispensed medicines per prescription and the travel cost borne by patients with NCDs to reach the facility.

The qualitative component of the study included FGDs with community members, health workers, PHC medical officers and pharmacists from private pharmacies, and in-depth interviews with PHC medical officers and district health officials. The FGDs among community members were conducted with the objective of understanding their experiences and identifying barriers to accessing care at PHCs, particularly for patients with diabetes and hypertension. The health worker FGDs with accredited social health activists (ASHA, the village level community health worker) and auxiliary nurse midwives (ANMs) captured their perspectives on care for diabetes and hypertension at PHCs. ANMs largely provide reproductive and child healthcare at subcentres, the first point of contact with government health services, and implement various disease control programmes. In the FGDs with private pharmacists, we explored their perceptions of medicine price and quality in public and private sectors and their perceptions regarding generic medicines. Details of FGDs conducted are provided in table 1.

**Table 1:** Details of FGDs

Number of FGDs	Group	Focus of discussion
5	Patients	NCD burden and care
2	Healthy adults (non-NCD)	Health-seeking behaviour, perceptions of quality of healthcare and medicines
3	Health workers	NCD burden and community awareness, NPCDCS implementation at subcentres
3	Medical officers	NCD care at PHCs including the challenges they face, NPCDCS programme activities
1	Private drug shopkeepers	Views on generic medicines for NCDs

FGD, focus group discussion; NCD, non-communicable disease; NPCDCS, National Programme for the Prevention and Control of Cancer, Diabetes, Cardiovascular Disease and Stroke; PHC, primary health centre.

The interviews with PHC medical officers focused on their experiences and challenges in delivering care for patients with NCD, including prescription and medicine dispensing practices. Apart from these, we used data from the process documentation done as part of the study. A trained observer participated in *taluka* level monthly staff meetings in the study *talukas*. Notes were taken on issues discussed especially around NCDs and their management. A total of 30 meeting reports and 36 field visit diary entries were analysed.

In this paper, qualitative data from FGDs, process documentation and in-depth interviews, quantitative data on healthcare utilisation and community perceptions of medicines from the household survey, and data on medicine availability and stockouts from the facility survey are used. The full details of the sampling design and tools used are available in the study protocol.^15^

### Data analysis

Quantitative data were entered into a spreadsheet format using EpiData software.^20^ Validation checks were conducted to find any errors in the data set. Transcripts of in-depth interviews and FGDs as well as field notes were imported into NVivo for coding.^21^ The FGDs, in-depth interview transcripts and meeting reports were coded and a thematic analysis done. We scanned transcripts and coded portions of text reflecting previously prepared codes; we also added codes that emerged in the process of analysing the transcripts. These codes were scanned by the research team and organised into themes. The codes and themes were examined with respect to their relationship with one or more health system components of the Bigdeli *et al* framework. Themes were identified across these codes and organised using the framework. Coding and identification of themes was done initially by the first author and was then discussed and finalised among coauthors.

**Figure 1:**
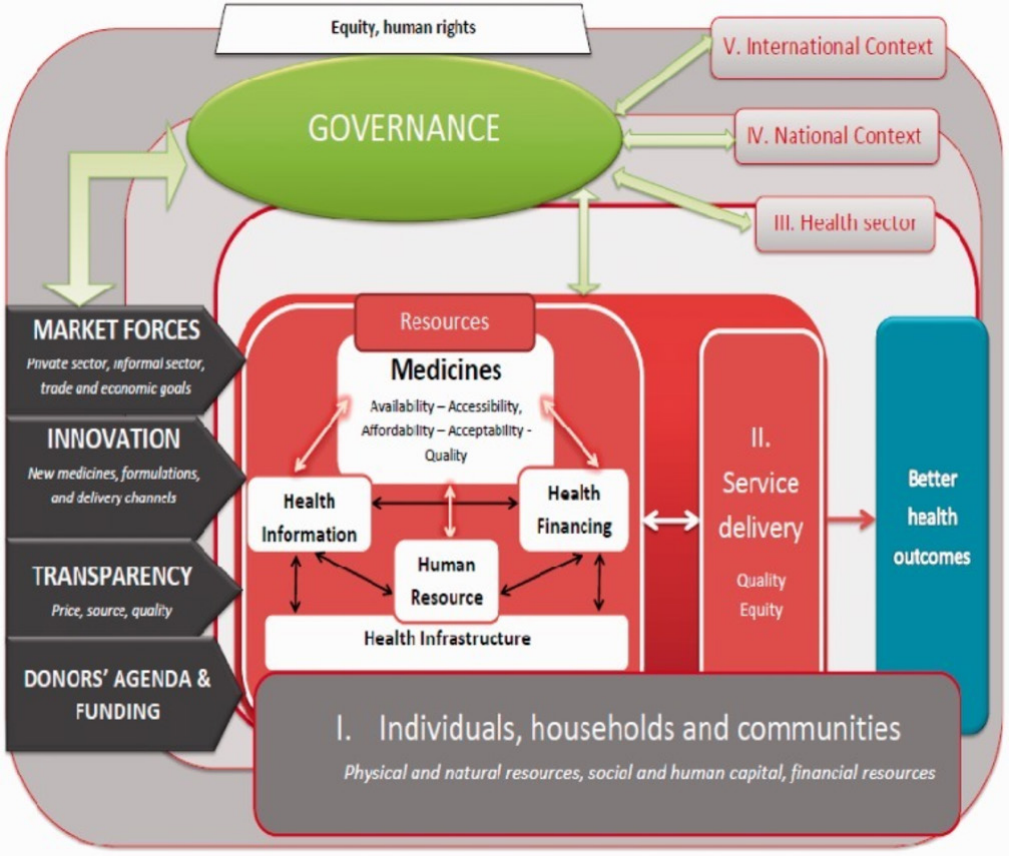
Access to Medicines from a health systems perspective (Bigdeli *et al* 16).

The Bigdeli *et al* Access to Medicines framework (figure 1) examines the demand and supply side constraints that influence access to medicines, and the dynamic relationships between all building blocks of the health system, and considers the leadership and governance of the health sector in their local, national and international contexts. The framework facilitates explanation of results at the local health system and health service levels in relation to wider systemic elements that affect health services and systems. In our study, given the emphasis on the district level, we limit our exploration to the local and national contexts and our analysis does not include other elements of the framework such as donor agendas and funding, that are not within the scope of our study.

### Ethical considerations

The study proposal received ethics approval from the WHO Ethics Review Committee and the Institutional Ethics Committee of the Institute of Public Health, Bangalore. The permission for data collection and facility surveys was obtained from the Department of Health and Family Welfare, Government of Karnataka. Informed consent was obtained for the survey, in-depth interviews and FGD respondents. Confidentiality of data was maintained by removing any identifiers from the data sets and transcripts.

## Results

The household survey sample included a total of 1149 patients with diabetes and/or hypertension across 1069 households. While 486 (42.3%) of these patients reported having hypertension and 395 (34.4%) reported having diabetes, 268 (23.3%) reported having both conditions. The major results from both quantitative as well as qualitative components of the study are presented in this section, under relevant themes, organised using the Bigdeli *et al* framework. The summary of the themes is provided in table 2.

**Table 2:** Main themes identified pertaining to NCD care as per the Bigdeli *et al* framework

Health system components	Main themes
Individuals, households and communities	Private sector dependence: More than 90% of respondents depended on private facilities for NCD medicines. An important driver seemed to be fragmented care in the public sector. Views on medicine availability: Poor availability of medicines at PHCs was reported to be the key reason for private sector dependence. Views on medicine quality: There is a perception that medicines supplied in the public sector are of inferior quality.
Resources: human resources and infrastructure	Shortage of health workers: 30% of PHCs did not have a doctor (due to a vacant post). Similarly, in 31% of PHCs, there was no full time pharmacist and in 39% of PHCs there was no laboratory technician posted. Poor laboratory facilities: Only 38% of PHCs had a functional laboratory (a designated area with basic laboratory equipment and a laboratory technician), where diagnostic tests related to NCDs (blood cholesterol and blood sugar levels) were conducted.
Health service delivery	Medicine availability at PHCs: More than 60% of PHCs reported more than 1 month of stockout of basic medicines for diabetes. Thirty per cent of PHCs reported more than 6 months stockout of metformin and 33% of PHCs had a stockout of glibenclamide (both drugs for diabetes) for more than 6 months. Only 5% of facilities reported availability of any statin at the time of visit. Inadequate primary care component of NPCDCS: The national programme is limited to sporadic screening camps in the community and other activities at the secondary and tertiary levels. At the PHC level, health workers had not received training in the management of diabetes/hypertension, nor were they given specific guidance on what aspects of care ought to be provided at the PHC level.
Governance	NCD prioritisation at district/taluka local health systems: The local health system agenda is strongly influenced by state and national programme priorities. NCDs do not appear among the top priorities for medical officers or health managers based on discussions during monthly review meetings. In review meetings and discussions, communicable diseases and reproductive and child health get most attention in terms of time spent on review and monitoring. Financial and managerial resource allocation for NCDs in general, and for diabetes and hypertension in particular is relatively low.
Market forces	Promotion of combination medicines by doctors and preference for combination medicines by patients. Private practitioners influence patients’ perceptions related to quality of medicines and/or care at government centres.
Transparency	Corruption and informal payments: Demand for informal fees for care at PHCs, setting up private practice either during work hours or diversion of PHC time/resources to private practice.

NCD, non-communicable disease; NPCDCS, National Programme for the Prevention and Control of Cancer, Diabetes, Cardiovascular Disease and Stroke; PHC, primary health centre.

### Individuals, households and communities

#### Private sector dependence for NCD care

Less than 10% of household survey respondents obtained their medicines from a government hospital or PHC. About 76% of patients depended on private pharmacies to obtain medicines. About 60% of households believed they could get free medicines from a government PHC. More than 90% of households believed that medicines were more expensive at private pharmacies and 75% of households reported that they could not afford medicine expenses. About 40% of families reported that they had to borrow money or sell assets to be able to pay for medication that they needed. Among all households, less than 3% reported that their medicines were covered under any kind of insurance scheme.

We explored the reasons for private sector dependence/preference in our FGDs. Although patients considered treatment in the private sector to be more expensive, they perceived the quality of care offered by private facilities to be better than that offered in public facilities. These perceptions seemed to be influenced by an expectation of continuous care. For instance, frequent changes in PHC doctors possibly explained multiple respondents’ view of inconsistency of care as a reason for choosing private facilities. Another possible driver of private sector dependence was the availability of integrated care in the private sector. All services required were offered under one roof although for a fee. A related barrier in government facilities was the need for multiple visits for consultation and diagnostics. In the case of poor households, this results in increased wage loss and higher indirect costs. This could be driving the choice of the private sector despite higher costs at point of care. To quote a few community members,

In government (facilities), the expenses will be less but the treatment is not fast. They do not check the patients as fast as possible. So we go to private hospitals.

FGD_com6

We go to government (facilities) for common ailments. If there is a specific doctor to be seen we will go elsewhere.like if we want to show to a skin specialist, we go to the private (doctor)… because the (government) doctor is not available when we need them, we go to private hospitals.

FGD_com1

When I asked them (government PHC laboratory) to tell me the sugar level, they said that they do not have the machine and asked me to get it tested outside. They told the same thing from the past 4–5 months.

FGD_com2

#### Views on Medicine availability

Poor and irregular medicine availability at PHCs emerged as an important reason for dependence on the private sector. Only about 38% of survey respondents felt that medicines for their condition were available in government facilities; whereas 96.8% reported that the medicines were available in nearby private pharmacies. The dependence on private pharmacies possibly enhanced the perception of poor care in the public sector, more so for patients with diabetes and hypertension, as they need long-term or lifelong medicines. The following two quotes from community members reflect this.

If we go to general hospital (government hospital), if we ask for 30 tablets they will give only 20, and if we come here (government PHC) the doctor will not be available, only compounder (pharmacist) will be there, he gives the tablets only if it is available otherwise he will say no, so for all these inconveniences what we do is we go to private (hospital).

FGD_com4

I have never gone to private (hospital) at all. From my birth, I have not gone to private (hospital). But, when I go to government (hospital), they say there are no drugs available and they will prescribe tablets (to be bought outside). Last time I asked them (government PHC) for BP tablets and they told there is no supply and prescribed for outside and I purchased from outside.

FGD_ com6

This was echoed in the following quote from a PHC medical officer:

People are more depending on private hospitals for medicine, whatever facilities government is giving (are) not reaching people and there is lapse from our side also. We have only basic medicines, not second-generation medicines. Now people take second-generation medicine for BP (hypertension) and diabetes so they are not coming to PHCs.

ID_MO1

#### Views on Medicine quality

People perceived the price of a medicine to be an indicator of its quality. We did not find any mention of reliance on statutory drug quality monitoring systems of the government for medicine quality among patients. One of the possible drivers of this perception could be a similar perception regarding price and quality among health workers (including doctors). Respondents shared that doubts about medicine quality in the public sector were also brought up during consultation in government facilities. In line with this perception from FGDs, we found that only 31% respondents in the survey felt that the medicines supplied in government facilities were of good quality. About 90% of the survey respondents reported that they felt if the medicine price was higher, it would be of better quality. Only 10 respondents (0.9%) had ever heard of generic medicines. The following quotes from community members explain this:

Yes, but it will take time, because they (government PHC) will have low quality tablets, so if we want to have original tablets we have to go to private clinics and bring and if we take that we will recover immediately.

FGD_com2

In the quote, the word original is reproduced without translation from Kannada. Used in this way in Kannada, it means both power/potency as well as authenticity (implying that a product described as *original* is not counterfeit/fake).

Everybody has their own opinion, for me they (government PHC) give 30 tablets per month I have to take it twice a day, and if I show to other doctor (in private) he says, the tablet given there is of less power, so your sugar is not coming to control.

FGD_com4

Doctors from the public sector pointed out that PHCs do not get combination medicines, which are popular in the private sector. Many doctors discussed the demand from patients for combination medicines, as they were hesitant about taking too many tablets. FGDs with private pharmacists confirmed this demand, although it was attributed to patient compliance. In the private sector, combination medicines are often prescribed in line with patient demand even where such combinations are not part of standard treatment guidelines or national programmes. The following quote by a health worker who reflected more on this issue illustrates this.

The supply we have is only Metformin and so, the private doctor will write the combination of both in one tablet, so the people think I can take only one tablet whereas in government they had given two tablets, it is happening like this.

FGD_HW4

This was further corroborated in the private pharmacist FGDs. In the words of a private pharmacist:

If you go to a doctor if he writes single (active ingredient per tablet), he has to prescribe eight tablets, the patient will say, the doctor has prescribed too much of (many) tablets, how to take it, if you write two tablets of four combination each, it will be easy for people to take and another thing, when they want to hike the price, some will have permission for combinations and not for single, so, manufacturers will think about all these things and wherever they get permission for multiple combinations, they will introduce those tablets (in)to (the) market.

FGD_Pvt Ph

### Resources: human resources and infrastructure

There is a shortage of doctors, pharmacists and laboratory technicians in the *talukas* selected for the study (table 3). Many of the PHC doctors are expected to manage more than one PHC. According to these PHC doctors, managing more than one PHC poses many challenges as they are expected to carry out administrative responsibilities at both PHCs along with clinical work. The shortage of pharmacists and other paramedical staff also poses challenges in providing required care at PHCs.

**Table 3:** Details of health personnel in the study *talukas*

Taluka	Number of PHCs	Percentage of PHCs with full time MOs	Percentage of PHCs with full time pharmacists	Percentage of PHCs with lab technicians
Taluka 1	11	36	81	45
Taluka 2	17	88	47	65
Taluka 3	11	81	91	73

PHC, primary health centre.

There are also infrastructural shortages. We found that of the 39 PHCs, only a few had a functional laboratory equipped to carry out the necessary blood tests for diabetes (table 4). As part of the NPCDCS programme, glucometer strips were supplied to PHCs, but during the study period, the supply was irregular. Many a time, the supplied strips were near their expiry date, due to which the facilities had to use them quickly within a few months of their supply.

**Table 4:** Details of laboratory facilities in the study *talukas*

Taluka	Number of PHCs	Percentage of PHCs having lab facilities	Percentage of PHCs having a functional lab	Percentage of PHCs having facility for NCD tests
Taluka 1	11	91	45	27
Taluka 2	17	88	65	29
Taluka 3	11	73	73	64

NCD, non-communicable disease; PHC, primary health centre.

These factors were further corroborated and explained by patients and PHC staff. According to PHC staff, due to the lack of paramedical staff and/or equipment, they had to refer patients elsewhere. To quote a community member:

Yes, now what is happening is the (government PHC) doctor will not be available till 11 or 12 in the morning, we have to wait there till the doctor comes and then when he comes, if he prescribes us for a scanning or any other higher tests, he usually refers us to the taluka hospital, because we do not have scanning facilities in here, so by the time we go there get the test done and come back, the doctor here would have gone home, so we cannot get ourselves a check-up in 1 day from morning till evening.

FGD_com1

We found that the distribution of resources (health workforce and infrastructure) was not uniform across *talukas*. This has been seen in earlier work at the district level wherein the administration at the taluka level and the general governance at the district and taluka levels are variable and often influence intervention implementation and outcomes.^22^

### Service delivery

#### Medicine availability at PHCs

We assessed medicine availability at PHCs from the study *talukas* using a checklist based on the essential medicines list for hypertension and diabetes. At the time of the survey, the most commonly available medicines at PHCs for hypertension were atenelol and amlodipine and for diabetes, metformin (table 5). Only about 5% of facilities had any statin in stock at the time of the survey.

**Table 5:** Details of medicines available at PHCs

S No.	Name of the medicine	Medicine included in EML, Karnataka (yes/no)*	Medicine included in NEML (yes/no)†	Availability checked at PHC (yes/no)	Number (%) of PHCs this medicine was available in during the visit (n=39)
1	Tab atenolol 50 mg	Yes	Yes	Yes	24 (61.5%)
2	Tab amlodipine besylate 5 mg	Yes	Yes	Yes	19 (48.7%)
3	Tab metformin 500 mg	Yes	Yes	Yes	17 (43.5%)
4	Tab glibenclamide 5 mg		Yes	Yes	2 (5.1%)
5	Tab atorvastatin 10 mg	Yes	No	Yes	2 (5.1%)
6	Tab enalapril maleate 10 mg	Yes	Yes‡	Yes	1 (2.5%)
7	Tab hydrochlorathiazide 50 mg	No	Yes	Yes	0
8	Tab losartan 50 mg	Yes	No	Yes	0
9	Tab pioglitazone 1 mg	Yes§	No	Yes	0
10	Tab glimepiride 1 mg	Yes	No	Yes	0
11	Tab methyldopa 250 mg	Yes	Yes	No	Not applicable
12	Inj insulin 40 IU/ml	Yes	Yes	Yes	1 (2.5%)
13	Inj premix (30:70) insulin 40 IU/ml	Yes	Yes	No	Not applicable

*Medicine included in the state essential medicine list 2014–2015.^29^

†Medicine included in the Indian NEML.^30^

‡NEML includes only 2.5 mg and 5 mg dosage for tablet enalapril maleate.

§Tab pioglitazone is included in the state essential medicines list but is supposed to be available only at secondary and tertiary centres.

EML, essential medicines list; PHC, primary health centre.

**Figure 2:**
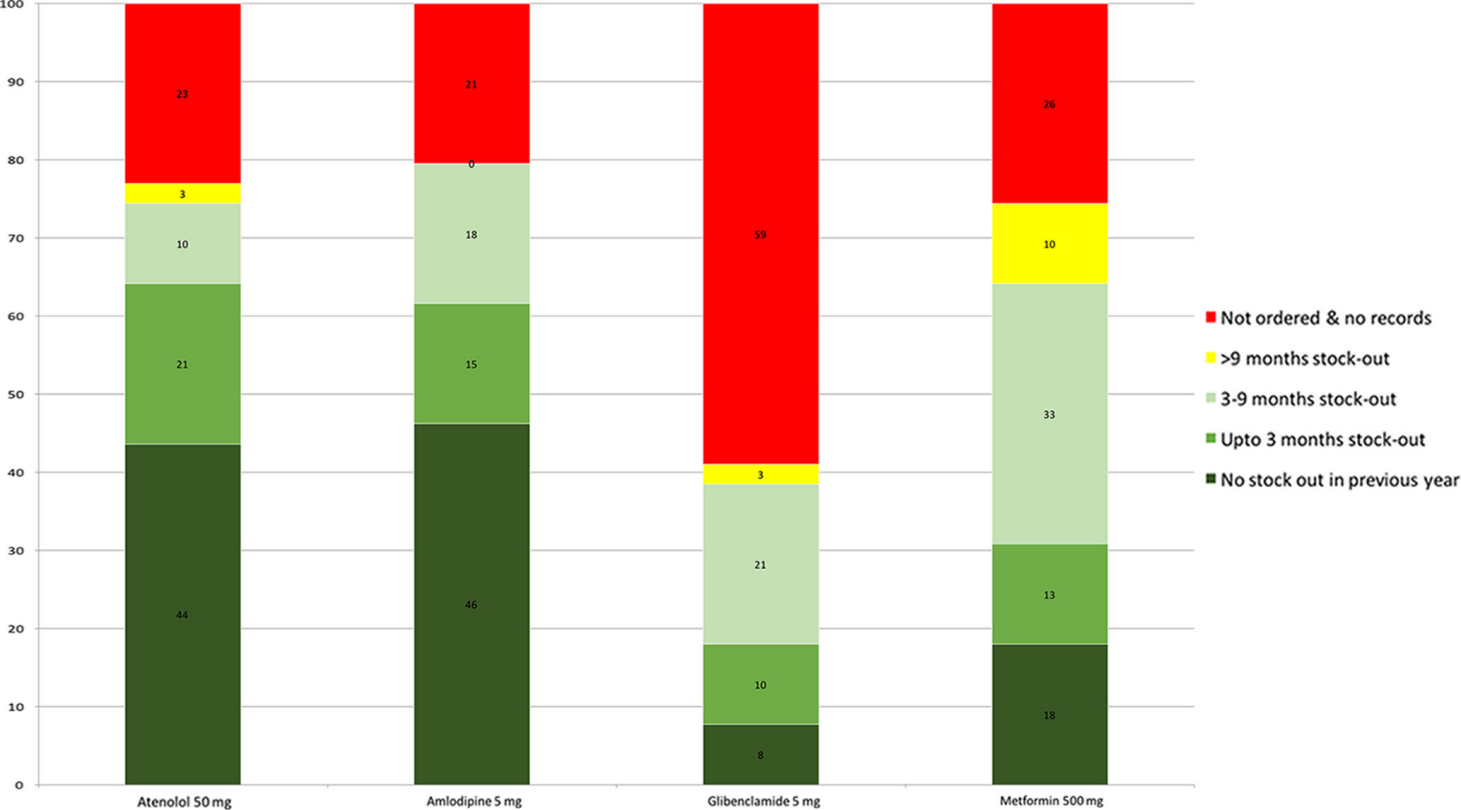
Details of stockout in study primary health centres (PHCs); numbers in the graph indicate percentage of PHCs.

We also assessed average period of medicine stockout^ii^ in the survey PHCs. We obtained stockout information for four NCD medicines, that is, two for diabetes (glibenclamide and metformin) and two for hypertension (atenolol and amlodipine). It was noticed that other diabetes and hypertension medicines commonly used in the private sector and mentioned in the state Standard Treatment Guidelines, such as enalapril, losartan, atorvastatin, pioglitazone, glimepiride and insulin were not procured by PHCs in the study *talukas* (figure 2).

We found that around half of the PHCs did not have stockouts of atenolol (44%) and amlodipine (46%). Nine PHCs (23%) did not place an order (and hence did not have any stock) for atenolol tablets and eight PHCs (21%) for amlodipine tablets. However, for antidiabetic medicines, over half of the PHCs (59%) never placed an order for glibenclamide, and 26% of them never placed an order for metformin. For glibenclamide, we found that only in three PHCs (8%) were there stocks throughout the year.

#### Inadequate primary care component of NPCDCS

Though the NPCDCS programme lists prevention and control of NCDs as one of its main objectives, we found that activities under the programme at the PHC level were limited. Though ASHAs are the first line of contact for the community, they had not received any training on diabetes/hypertension or their role in the management of diabetes/hypertension. Many ASHAs we interacted with were not familiar with any non-drug treatment approaches to diabetes/hypertension (lifestyle modification, diet management or specific counselling points for NCDs).

We found that screening camps are organised for several NCDs at the PHC level; but they are *ad hoc* and isolated in their implementation without specific linkages to ongoing PHC or NPCDCS activities. Health workers reported that once they conduct screening camps, people put pressure on them to reorganise such camps. A health worker reflected:

They come and ask us when shall I come for the sugar test, should I come fasting, when will you hold the camp, you were doing it for some days and now you have stopped, they ask all these questions.

FGD_HW1

Health workers often get a limited supply of materials such as glucometer strips, and they could not conduct the targeted number of camps. To quote a health worker,

The strips got over, so I thought I will buy myself and I have called the (main district level) pharmacy so many times and asked them give us the strips of glucometer

FGD_HW3

Yet another issue highlighted by health workers was that after conducting screening camps, patients had to depend on the private sector or were referred to *taluka* hospitals for medicines and continuous care, as medicines were not usually available in PHCs.

Six months back, the sister (nurse) had checked the BP (hypertension) and sugar (diabetes), there (were) no tablets, so what the people say when we call them is- she will not give tablets, she only checks the BP, so why should we come for that.

FGD_HW2

### Governance

#### Lack of prioritisation of NCDs

We found that NCDs are often not perceived as a priority by many health workers and managers. They tend to prioritise communicable diseases and maternal health problems over several NCDs (observations based on the length of time spent on review and discussion in monthly review meetings of health workers). They attributed the general lack of medicines for diabetes or hypertension to poor prioritisation at the district and higher levels. Non-drug treatment options especially counselling for patients with diabetes or hypertension did not come up in the course of planning and discussion. Among doctors, the perception that communicable diseases (unlike NCDs) need an urgent response could be driving this. The following two quotes show the views of PHC medical officers:

But if a person gets H1N1 (influenza) the patient dies, isn’t it? If a person gets BP (hypertension) or sugar (diabetes) he or she can live some more years, may be his life span will decrease by 5 years that is all. But H1N1 patient dies immediately, BP or sugar patient live without medicine for more than a year.

ID_MO2

We have to keep stock of NCD drugs, but in meetings (at taluka level) they insist us on purchasing some emergency drugs like fluids for dehydration patients in summer and dog bite medicine etc.

ID_MO8

From the observations of monthly review meetings, we found that NCDs get less attention compared to maternal and child health or communicable diseases at the *taluka* level. There is limited discussion on NCDs or medicine stockouts at review meetings at district and subdistrict levels. The only NCD related discussions that featured during the study period were announcements about screening camps for diabetes or cancer conducted by non-governmental charitable organisations or government superspecialty centres. We explored the reasons for this in our interviews with medical officers. A medical officer shared his thoughts on this:

They (in review meetings) will think more of immediate cause of death, meaning, the death will be fast because of acute diseases, chronic diseases means, we can take care of it next month, again next month, we postpone like that, so this happens. And one more thing, here, we see very less deaths, because of the chronic diseases, it is very less, usually those are seen in corporate based hospitals and higher hospitals, the treatment is done there, so, they will be seeing the deaths more, in our centres, we just refer, so we do not take note of it much.

ID_MO9

Training related to NCDs too receives less attention compared to other programmes; only a few pharmacists had received training in managing diabetes or hypertension in the study *talukas*. Others had not attended any orientation or training programmes on NCDs. The training organised as part of our research intervention was the first ever training session on diabetes or hypertension that many health workers and ASHAs had attended.

### Market forces

We found that the private sector plays a major role in influencing people’s choice of healthcare as well as medicines. Patients as well as health workers narrated instances where private health providers explicitly discouraged patients from using medicines provided in the public sector and persuaded them to purchase medicines from private pharmacies. Patients reported that even staff from government facilities advise patients to go to private facilities. In their words,

When they suggest particular doctor, both will have some mutual understanding. They just write a slip (prescription) and ask us to go buy outside medical shop and from there we have to come here and get the treatment done. I have seen with my own eyes and the government hospital people suggest us to go to private hospital. For all the medicines, we have to go to medical shop. If we ask them for glucose, they say that it is not there and if we ask them to check properly, they bring one bottle and say that it was there somewhere and ask us to pay Rs.70/- or Rs.80/-.

FGD_com6

### Transparency

We found that government doctors working in the private sector during working hours (in Karnataka, private practice after the duty hours is permitted) is common, often driving patients to private hospitals. Another common practice among doctors is prescribing medication that must be purchased from private pharmacies, sometimes due to non-availability of medicines or due to demand for particular medicines. This places a financial burden on patients with hypertension and diabetes, as they need to come for repeated follow-up visits and need to be on long-term medication. A community member reflected on the availability of doctors in PHCs:

Actually, she (government PHC doctor) will be in private hospital in the morning it seems, I came to know that she will go to government hospital after 12 noon, so once I went after 12 in the noon to the government hospital and showed there, she treated me for free, the same doctor in the private hospital will charge us to treat.

FGD_ com1

Community members and health workers pointed out that even when the medicines are available in PHCs, doctors hesitate to prescribe those. They prefer to send the patients to nearby private pharmacies irrespective of the medicine stock status. The following quotes by the health workers and community members reflect their experiences:

They (government PHC doctor) will not give the medicines, they will keep the stock, till today we do not know why do they hold the stock, and after it expires they will burn it… they have given me expired tablets to use as firewood to boil the water and I have done it. This is my experience, this has happened (to) me after joining as ASHA worker, I have brought two to three boxes and burnt (them).

FGD_HW1

Worst is…, doctor…, and he is good also, but they will not even give a cough tablet or syrup even for children, everything they prescribe (from) outside, there is a medical shop next to the centre and they both are friends, there will be so many tablets on the table of the doctor but he will not give a single tablet to anybody.

FGD_ com1

## Discussion

We found that there are many barriers at community and health service levels that influence access to medicines for diabetes and hypertension. Community perceptions of public sector medicine quality and poor medicine availability at PHCs appear to be reinforcing prevailing private sector dependence, especially in the case of diabetes and hypertension. This adverse perception in the community could undermine efforts to improve access to medicines in public hospitals. At the same time, public sector investment in terms of improving governance and administration, as well as improving medicine supply chains to local health systems at the district and subdistrict levels in India appear to be important precursors before any vertical programme or state-led efforts can be taken up for improving care for NCDs at the primary healthcare level. Below, we discuss findings from our study that are relevant to efforts for improving care for diabetes and hypertension at the primary healthcare level in India.

### Prioritising NCDs within primary healthcare

Since the national programme (NPCDCS) for improving care for diabetes and hypertension was being implemented during our intervention, the study provided an opportunity to assess the implementation of this programme, in relation to access to medicines. However, programmes such as these devised at the national level need to work through leveraging the same resources at PHCs and districts that are available to all other programmes and in this sense, they compete for financial and human resources with other programmes. Though the NPCDCS identifies the need for a comprehensive management strategy for NCDs at primary care, including risk reduction, prevention and early diagnosis, it does not specify any particular activity to be conducted at PHCs. On the other hand, its focus on organising screening camps, without much attention to continuous care, follow-up and other required services for long-term management of NCDs at PHCs, needs to be revisited. Given the widespread criticism of vertical approaches towards disease control, many lessons could be learnt from how the tuberculosis control programme went from being a national programme to generating greater ownership at the district and PHC levels.^23–25^ Our results indicate a dichotomisation within the health system wherein PHCs are expected to organise immunisation, reproductive and child health services and infectious diseases response while chronic health conditions are supposed to be addressed at tertiary and superspecialty hospitals.

### Governance and financing of a mixed public-private health system

Our assessment highlights the poor situation of access to medicines and stockouts. The lack of availability of medicines in government facilities poses a huge challenge for NCD management. Frequent stockouts of NCD medicines result in overdependence on the private sector, which in turn increases out-of-pocket expenditure and impoverishment.^7 26^ In addition to medicines, the clinical management of NCDs—until it reaches a very advanced stage—rests on long-term and continuous care that tests health systems (the supply of medicines, motivation of health workers to go beyond an immediate crisis, organising health promotion and options for lifestyle modification, frequent follow-up even when otherwise healthy and awareness building on the part of community health workers, and governance of the pharmaceutical industry). Various health system issues especially primary healthcare infrastructure, availability of human resources and capacity and investments in creating a reliable and responsive supply chain for medicines need to be addressed before PHCs in Tumkur and possibly in many parts of Karnataka can provide NCD care.

Given the lack of a universal health coverage system in India and the high share of medicines in OOP expenditure, complications arising from NCDs could be significant contributors to household health expenditure, often at the point of service delivery. In our study, less than 3% of respondents reported any coverage of insurance for NCD medicines. 8 27 28In fact, private health sector dependence may be more in NCDs than in other conditions where the public sector, particularly at the primary care level, gives greater attention (such as reproductive and child health or immunisation). Our study findings related to the stockout of medicines and private sector dependence for NCD medicines acquire a greater importance given the expanding private sector in health in India. This is happening within an environment where regulation of the private sector by states is lacking on one hand, and on the other, coverage for NCD-related services are woefully limited (confirmed by our study as well). Policy responses for improving care for diabetes and hypertension in the public sector will in turn need to consider issues around private sector regulation. Lack of action over the years may reinforce private sector dependence and diminish overall public health system credibility in the long run.

### Prioritising NCDs in India’s federal structure

There is a need to re-frame NCDs as diseases that are not limited to affluence, as is evident from various studies across the country.^8 27^ The traditional focus in most Indian states on reproductive and child health and communicable diseases, while certainly needed, should not come at the cost of compromising care for other conditions. Moreover, it is important to focus on PHCs as points of delivery of comprehensive primary healthcare as opposed to centres for maternal and child health or for infectious diseases. Currently, NCD care at PHCs suffers from this wider public perception, and is in line with how health workers themselves view their role.

While the NCD agenda has been powerfully set at the global level through the UN general assembly resolution and various other global and transnational institutional arrangements focusing on NCDs, local health system acknowledgement of the epidemiological transition appears to be limited. Further, in India under the constitutional arrangement, health is a state subject and Indian states vary in their organisation, financing and management of health services. Decentralisation to district level has been advocated and is being implemented. Despite such decentralisation on paper, several districts in Karnataka have not taken up the increased role envisioned for them and hence there is an assumption that districts will steer their own agendas based on a local situation analysis, when in fact the capacity for this has not been systematically built up.^22^ There is a need for innovative approaches that recognise the continuing need for support from above while building local capacities at the district and subdistrict levels, in line with the decentralisation of health planning and increased financing proposed in the National Health Mission.

## Footnotes

i A cluster here means a group of households (typically in a single village) within the catchment area of a PHC.

ii Stockout period refers to the number of days that a medicine was not available at a surveyed facility.
